# Gastrointestinal manifestations related to infection with SARS-COV-2: Appendicular syndrome (A case report)

**DOI:** 10.1016/j.amsu.2021.102288

**Published:** 2021-04-14

**Authors:** Abdelilah Elbakouri, Abdessamad El Azhary, Mounir Bouali, Fatimazahra Bensardi, Khalid Elhattabi, Abdelaziz Fadil

**Affiliations:** a Department of Visceral Surgical Emergency (P35), UHC Ibn ROCHD, Casablanca, Morocco; b Medicine and Pharmacy Faculty, University of Hassan II, Casablanca, Morocco

**Keywords:** Appendicitis, CT Scan, COVID-19, SARS-CoV-2

## Abstract

**Introduction:**

The clinical symptomatology of SARS-CoV-2 disease may manifest as an appendicular syndrome. The abdominal CT scan can be used to rule out or confirm the diagnosis of acute appendicitis and a chest CT scan can make the diagnosis of SARS-CoV-2 infection.

**Observation:**

We report the observation of a 30-year-old patient, with no particular pathological history, who presented with appendicular syndrome without extra-digestive signs, and especially, without respiratory syndrome. The CRP was at 35mg/l. A thoracoabdominal CT scan was requested to detect an eventual appendicitis. With three straight frosted glass areas on the thoracic area suggesting COVID 19 infectious pneumopathy, a PCR was requested to detect a positive SARS-CoV-2 viral RNA, then the patient was appendectomized. Post-operative follow-up was simple and the patient was transferred to a department dedicated to covid-19-positive patients for further management.

**Conclusion:**

CT scan is necessary before considering emergency surgery for acute appendicitis because it can change the patient's management circuit.

## Highlights

• The Covid-19 Pandemic IS a major global health problem.
• SARS COV 2 infection can manifest itself in different clinical pictures.
• Though respiratory symptoms predominate gastrointestinal complications from SARS COV 2 infection have also been described and may even precede respiratory symptoms.
• In this time of pandemic it is important to consider this diagnosis even in cases of abdominal pain syndrome suggestive of acute appendicitis.
• A C T scan is necessary before considering emergency surgery for acute appendicitis.

## 1 Introduction

Since first being detected in December 2019, the novel severe acute respiratory syndrome coronavirus 2 (SARS-CoV-2) has spread worldwide and has been classified as a global pandemic of international concern [1]. The COVID-19 clinical presentation ranges from asymptomatic individuals or mild flu-like symptoms, to a very severe condition with interstitial pneumonia and acute respiratory distress syndrome (ARDS) [2]. While the pulmonary system is most commonly affected, extrapulmonary organs and organ systems (including the cardiac, gastrointestinal, hepatic, renal, ocular, and dermatologic) are also affected by COVID-19, which could have significant health consequences [3].

### 1.1 Observation

**Fig. 1:**
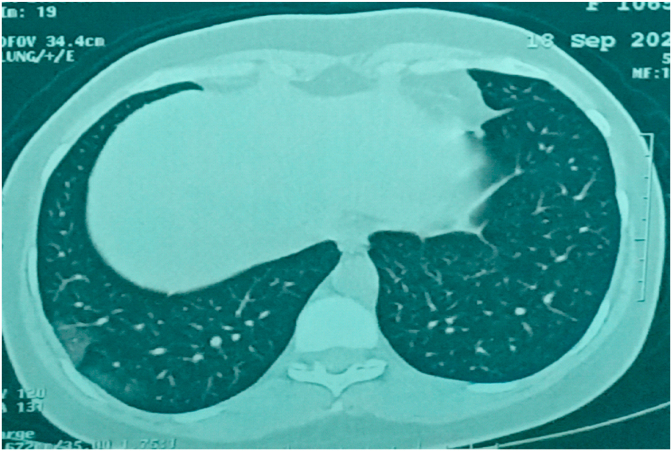
Abdominal pelvic scan. Swollen retro-caecal appendix measuring 10.1mm maximum diameter with grease infiltration opposite (arrow).

**Fig. 2:**
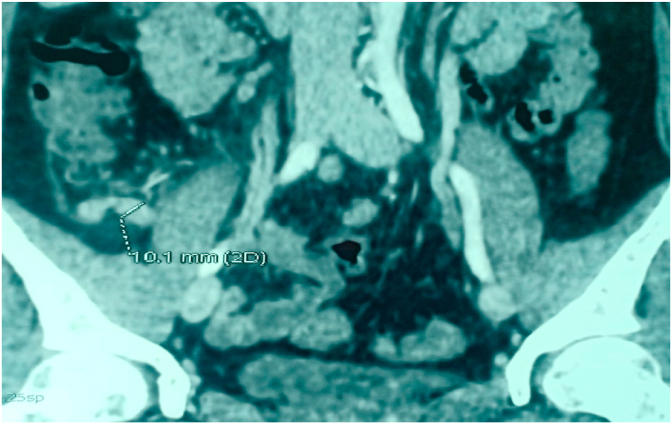
Chest Scan objectivizing Frosted Glass Opacity Images, Highly. suggestive of COVID-19 disease.

**Fig. 3:**
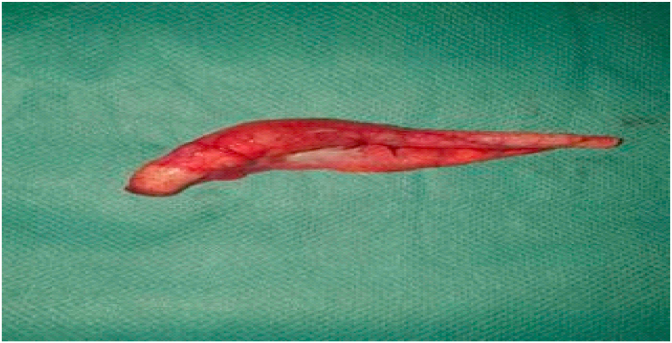
Intraoperative image of an inflamed swollen, bulging appendix.

This is a 37-year-old patient, with no particular pathological history, who presented with pain in the right iliac fossa, which had been evolving for 48 hours before her admission, without vomiting and without transit disorders, in a context of a febrile sensation and preservation of the general state of health. On physical examination, the patient was found to be stable on the hemodynamic and respiratory level with a febrile state at 38.3°. McBurney's sign was positive. No extra-digestive signs were reported, and in particular, no respiratory syndrome. The biology objected a moderate inflammatory syndrome with CRP at 35mg/l and WBC at 12,580/mm3 (absence of leukopenia and lymphopenia). A thoracoabdominal CT scan was requested and detected a swollen retrocaecal appendage measuring 10 mm maximum diameter with fat infiltration opposite (Fig. 2). With three straight frosted glass areas on the thoracic area suggesting COVID 19 infectious pneumopathy (Fig. 1), a PCR was requested to detect a positive SARS-CoV-2 viral RNA, then the patient was appendectomized (Fig. 3). Post-operative follow-up was simple and the patient was transferred to a department dedicated to covid-19-positive patients for further management. the patient was followed in consultation with good evolution.

## 2 Discussion

The SARS-CoV-2 is an enveloped, single-stranded RNA virus that can be transmitted from human to human [4]. Bats have been identified as a key reservoir of coronavirus in China. The SARS-CoV-2 is about 50% genetically identical to MERS-CoV and about 79% identical to SARS-CoV, to which it has a similar receptor-binding domain structure [5]. Due to the novelty of the virus and the short duration of the SARS-CoV-2 outbreak, only a limited and scattered body of scientific evidence is available on various aspects of COVID-19 [6]. SARS-CoV-2 infection can manifest itself in different clinical pictures [7]. Though respiratory symptoms predominate, gastrointestinal (GI) complications from SARS-CoV-2 infection have also been described, and may even precede respiratory symptoms [8]. Of note, 10% of patients experienced nausea and diarrhea between 1 and 2 days before experiencing respiratory symptoms, suggesting that GI symptoms may atypically present as one of the initial clinical manifestations of COVID-19 [9]. The fecal-oral route has been proposed as a potential mechanism of GI infection with SARS-CoV-2 [10] due to the identification of SARS-CoV-2 RNA in the stool specimens of infected patients [11]. Xiao et al. studied the RNA in feces from 73 patients with COVID-19, and 53% of the patients tested positive for SARS-CoV-2 RNA in the stool [12]. Additionally, studies have found overexpression of ACE-2 in the epithelial cells of the GI tract, suggesting SARS-CoV-2 replication in the GI tract [13]. At this moment of the health crisis related to COVID-19, it is important to think about this diagnosis even in the case of symptoms suggestive of acute appendicitis. Before emergency surgery, we should look for a co-infection that can change the patient's management circuit and may be the cause for the therapeutic proposal to be reviewed.

## 3 Conclusion

The initial clinical presentation of coronavirus disease 2019 may be appendicular syndrome. An abdominal CT scan ruled out the diagnosis of appendicitis and a chest CT scan yielded a diagnosis of SARS-CoV-2 infection. CT scan is required before considering emergency surgery for acute appendicitis.

## Provenance and peer review

Not commissioned, externally peer reviewed.

## Sources of funding

None.

## Ethical approval

I declare on my honor that the ethical approval has been exempted by my establishment.

## Consent

Written informed consent for publication of their clinical details and/or clinical images was obtained from the patient.

## Author contribution

Elazhary Abdessamad: Corresponding author writing the paper and operating surgeon.

Bouali Mounir: writing the paper.

El Bakouri Abdelilah: writing the paper and operating surgeon.

El Hattabi Khalid: study concept.

Bensardi Fatimazahra: study concept.

Fadil Abdelaziz: correction of the paper.

## Guarantor

DR Elazhary Abdessamad.

## Declaration of competing interest

The authors declare that they have no links of interest.
